# Dr. Dowler on Death

**Published:** 1850-06

**Authors:** 


DR. DOWLER ON DEATH.

Dr. Dowler, of New Orleans, has attracted some attention, this winter, by his papers on the vivisection of the Southern saurian. The experiments were repetitions of a suite previously performed by Professor Le Conti, of Georgia, who has recently reclaimed them. Dr. Dowler has made a satisfactory response, in the last number of the New Orleans Medical Journal, to the Professor's complaint. In a late number of the New Orleans Medical Journal, the editor announced that Dr. Dowler was then engaged on a paper that would be likely to create a sensation. Through the courtesy of Dr. Dowler, we have received a copy of the paper referred to, and we have read it with some degree of pleasure, but with less satisfaction than we anticipated. The paper is entitled, "Researches on the Natural History of Death," and it contains not only a review of the action of the French Academy on what is called the Manni prize, but Dr. Dowler's own experiments and deductions, on the test of death. In order to understand the subject, the reader must bear in mind, that in 1837, "Professor Manni, of the University of Rome, proposed a special prize of 1500 francs, to be awarded by the French Academy, for the best work upon the subject of apparent death; with the view of preventing premature interments."—This prize was carried off by M. Bouchut, and he divides "the certain signs of death into *immediate* and *remote*. The first consists in: 1. The prolonged absence of the signs of the heart; 2. The simultaneous relaxation of the sphincters; and, 3. The sinking of the globe of the eye, with loss of transparency of the cornea. The first of these alone, is regarded by the committee as conclusive. The remote signs are: 1. Cadaveric rigidity; 2. The absence of muscular contractility under the influence of galvanism; and, 3. Putrefaction."

The first of the *immediate* signs of death, thus laid down by M. Bouchut, "cannot," according to Dr. Dowler, "be a general test, because stethoscopists are rare, and in the best hands is often not attainable." This does not seem to us a valid objection; if the test offered by M. Bouchut is the best one, that gentleman, backed by the authority of the eminent body who were appointed to judge the case, is clearly entitled to the prize. That which is the surest test, may not always be the most easily reduced to practice, while that which is most attainable, may not be the best. Take, for example, Dr. Dowler's "improvement" on M. Bouchut's test—the use of thermometers. Dr. D. considers that they afford a more unerring indication of the presence of death, than the absence of sounds in the heart. Would Dr. Dowler consider his own test fairly objected to, by the declaration that there were places where thermometers could not be readily obtained, and that they might fall into the hands of persons who did not know how to use them? This is precisely the character of Dr. Dowler's objection to M. Bouchut's test.

But again:—Dr. Dowler says: that the Academy and M. Bouchut are guilty of, what logicians call, *petitio principii*—"they take for granted what should be proved," and Dr. D. asks triumphantly: "Who has proved that the heart, like the pulse, like every other organ, may not fall into *temporary quiescence or inaction?*" Analogies are urged to show that the heart does fall into this condition, and saurian facts are referred to, which establish that a heart may act after it is cut out of the body: thus, an alligator's heart may be taken from his body and emptied of its blood; the alligator may then be roasted, the heart put back, and upon the application of the stethescope, "the dead will afford M. Bouchut's certain sign of life!"

With all due deference to Dr. Dowler, we think this is rather worse than what he charges on the French Academy and M. Bouchett—they only begged the question; Dr. Dowler changes its terms. M. Bouchut's test is founded on the *sounds* of the heart; Dr. Dowler undertakes to represent that it is based on the *movements* of the heart. The first sound of the heart may be admitted a muscular one, though we do not believe it, but about the second there can be no controversy—that is *valvular*, and may be produced by the passage of blood, or the constrained passage of air, moving both through the auricles and ventricles. Dr. Billing, in his admirable work on "The Principles of Medicine," shows that *both sounds are valvular;* Mr. Brakyn, of Trinity College, Dublin, has demonstrated the truth of Dr. Billing's views beyond all contradiction, and as these sounds are *valvular*, what force does Dr. Dowler find in his facts of muscular contraction in the exsected part? And how can he consider M. Bouchut and the French Academy antagonistic to his roasted alligator, and a heart emptied of its blood? Dr. Dowler does not invalidate the integrity of M. Bouchut's test, by his reasoning or assumptions. Mr. Brakyn represented recently that Dr. Stokes, of Dublin, had said he had "noticed in typhus fever, that patients, after the fever had progressed some time, and great debility had supervened; ceased to have any sound of the heart, though circulation continued. Sometimes, however, *the second continued*, the first being absent," &c. But Dr. Stokes has made a reclamation in the London Lancet for April, and says:

"I have shown that in a few cases a returning impulse is perceptible before the development of the first sound; but the statement that the heart may act with considerable energy for days, without producing any sounds, is altogether opposed to fact."

In our judgment, M. Brachat, in an article inserted in the Gazette Médicale de Lyon, has made a more philosophical assault on M. Bouchut's "infallible sign," than this of Dr. Dowler's. M. Brachat establishes, very conclusively, we think, the following facts, and they are serious obstructions to the reception of M. Bouchut's chief test:

"The survival of the organic over the cerebral life, may be manifested in other ways than by the contractions of the heart. Hemorrhages have occurred, sweatings continued, and the beard sprouted, after the cessation of cerebral life; and from the fact of the capillary circulation sometimes thus continuing, M. Brachat believes that we may discover a sign of death at least as important as that derived from the auscultation of the heart's action. As long as this circulation continues, ganglionary death has not taken place, and there is a possibility of the organic life reacting on the suspended cerebral life, and restoring it. The means of acquiring a knowledge of whether the capillary circulation does thus continue, consists in pricking a part abounding in capillaries, as the lips or the tongue. A droplet of blood presents itself if the circulation persists, which is never the case when life is extinct. This has often been verified in cases of asphyxia and apoplexy. For the same reason, as long as boiling water produces phlyctenæ, and mustard excites the redness of the skin, life is not entirely extinct in the exhalants and capillaries.

"Thus the two lives may be separately extinguished; the one surviving a while the other, especially the ganglionary. The most enduring actions are the contractions of the heart, the capillary circulation, the secretion of the skin, the increase of the hair."

Dr. Dowler undertakes to commend the use of thermometers as a test, superior to any that have been named under the Manni prize. This test assumes that nothing is taken for granted, without the most indubitable proof. The axiom is declared, that "man, in his living state, maintains an uniform temperature, independent of the surrounding media, while a dead man, like other inert matter, has no independence of this kind, but steadily responds to, and is governed by, calorific conditions altogether physical," &c.

We think Dr. Dowler assumes a great deal in this proposition. He begs the whole question with quite as much mendicity as the French Academy and M. Bouchut do. May we not ask Dr. D. his own question? "Who has proved that the heat-generating function may not fall into temporary quiescence or inaction," as well as the heart? We should like to hear this question answered.

Dr. D.'s paper has not been drawn up with the care it deserved. Passing over other blemishes, we venture to ask why Dr. Dowler permits himself to use such language as the following, which we find on page 20: "blood that *has underwent* refrigeration." Such things are spots upon the Doctor's reputation as a writer, and he should be more careful in future.

But we must bring these remarks to a close, although there are other portions of the Doctor's essay that require notice.

